# Inferring interaction type in gene regulatory networks using co-expression data

**DOI:** 10.1186/s13015-015-0054-4

**Published:** 2015-07-08

**Authors:** Pegah Khosravi, Vahid H Gazestani, Leila Pirhaji, Brian Law, Mehdi Sadeghi, Bahram Goliaei, Gary D Bader

**Affiliations:** School of Biological Sciences, Institute for Research in Fundamental Sciences (IPM), Tehran, Iran; The Donnelly Centre, University of Toronto, Toronto, Canada; Institute of Parasitology, McGill University, Montreal, QC Canada; Department of Biotechnology, College of Science, University of Tehran, Tehran, Iran; Department of Computer Science, University of Toronto, Toronto, Canada; National Institute of Genetic Engineering and Biotechnology, Tehran, Iran; Department of Bioinformatics, Institute of Biochemistry and Biophysics (IBB), University of Tehran, Tehran, Iran

**Keywords:** Gene expression data, Information-based approach, Interaction type, Regulatory interaction

## Abstract

**Background:**

Knowledge of interaction types in biological networks is important for understanding the functional organization of the cell. Currently information-based approaches are widely used for inferring gene regulatory interactions from genomics data, such as gene expression profiles; however, these approaches do not provide evidence about the regulation type (positive or negative sign) of the interaction.

**Results:**

This paper describes a novel algorithm, “Signing of Regulatory Networks” (SIREN), which can infer the regulatory type of interactions in a known gene regulatory network (GRN) given corresponding genome-wide gene expression data. To assess our new approach, we applied it to three different benchmark gene regulatory networks, including *Escherichia coli*, prostate cancer, and an in silico constructed network. Our new method has approximately 68, 70, and 100 percent accuracy, respectively, for these networks. To showcase the utility of SIREN algorithm, we used it to predict previously unknown regulation types for 454 interactions related to the prostate cancer GRN.

**Conclusions:**

SIREN is an efficient algorithm with low computational complexity; hence, it is applicable to large biological networks. It can serve as a complementary approach for a wide range of network reconstruction methods that do not provide information about the interaction type.

**Electronic supplementary material:**

The online version of this article (doi:10.1186/s13015-015-0054-4) contains supplementary material, which is available to authorized users.

## Background

With increasing amounts of biological data generated by modern high-throughput technologies, we are faced with a challenging problem: how to extract meaningful information from the data. A prominent direction for addressing this problem is using computational data mining approaches for the analysis of high-throughput biological data, such as gene expression data [1–4]. In particular, analysis methods have been developed to infer regulatory interactions from transcriptome data [5–14].These regulatory interactions link regulators, such as transcription factors and kinases, to their targets and may include the regulatory type of the interaction, which indicates whether there is an activating (positive) or inhibitory (negative) association between the interactor pair. Knowing the interaction type can be beneficial for a wide range of analyses including module-centric analysis [15] and network simulation [16]. A growing number of approaches use co-expression measures, either correlation-based (generally linear) or information theory-based (can consider non-linear relationships) [17], to infer GRNs.

Although information theory-based approaches have been widely applied to decipher GRNs [18–20], they are not currently used to determine the type of the regulation between two connected genes in a reconstructed GRN. Here, we present SIREN, a statistical framework that uses a new information theory-based measure to predict regulatory type. Our novel framework is capable of accurately predicting the type of regulation between two interacting genes. The fundamental assumption in our approach is that if two connected genes in the network have similar expression patterns, there is likely an activating (positive) association, among them. On the other hand, if their expression patterns are anti-correlated, the interacting genes likely have an inhibitory (negative) influence on each other. SIREN uses a mutual information-based measure to predict the interaction type. Extending mutual information has been extensively used as a similarity measure for feature selection fields [21–24]. In our novel approach, a rescaling matrix was introduced to convert the MI function, which normally generates non-negative scores, to a function that can have negative values. The resulting sign is used to predict the interaction type. While SIREN detects the regulation type, it cannot detect the direction of regulation. We evaluated SIREN by testing it on *E. coli*, prostate cancer, and in silico GRN benchmarks. In each case, SIREN reliably identified positive and negative regulatory types. Besides, comparison of SIREN with a baseline method based on Pearson coefficient correlation (PCC) revealed that it has a greater performance on biological GRNs. The R implementation of the algorithm is freely available at http://baderlab.org/PegahKhosravi/SIREN.

## Methods

### Information theory based metrics

Mutual information (MI) is a measure of the information dependency between two random variables, defined as:

$$MI = \sum {p(x,y)\log (p(x,y)/p(x)p(y))}$$

where *p*(*x*,*y*) is the joint probability of *x* and *y* and *p*(*x*) and *p*(*y*) are marginal probabilities.

If we define the function $f(x,y) = \log (p(x,y)/p(x)p(y))$, then $MI = \sum {p(x,y)f(x,y)}$ which is equivalent to the expected value of function *f*(*x*,*y*), i.e., $MI = E(f(x,y))$. $f(x,y)$ is defined to be the point-wise mutual information (PMI) [25]. PMI is a measure of how much the joint probability of a particular co-occurrence of events, *p*(*x*,*y*), differs from the expected joint probability, assuming the independence of *x* and *y*, *p*(*x*)*p(*y*) [25].

### Discretization of continuous data

For a computationally feasible calculation of MI and PMI, discretizing and binning of the expression data is required. In conventional binning approaches, each data point is assigned to exactly one bin. This can be problematic for data points close to the margins between bins: small, noisy fluctuations may cause these data points to be improperly assigned to a neighboring bin. Additionally, the choice of bin size can strongly influence the MI values for datasets of moderate size [26, 27]. To address these problems, we used a B-spline approach for data binning [27–30] that allows each data point to be assigned to multiple bins. To accomplish this, the indicator function, which typically maps each data point to a specific bin, was improved to allow each data-point to be assigned to several bins, with weights obtained from a B-spline function summing to one (schematically shown in Additional file 1: Figure S1). The B-spline function has a spline order that defines the shape of the function and influences the number of bins to which each data point is assigned.

**Figure 1:**
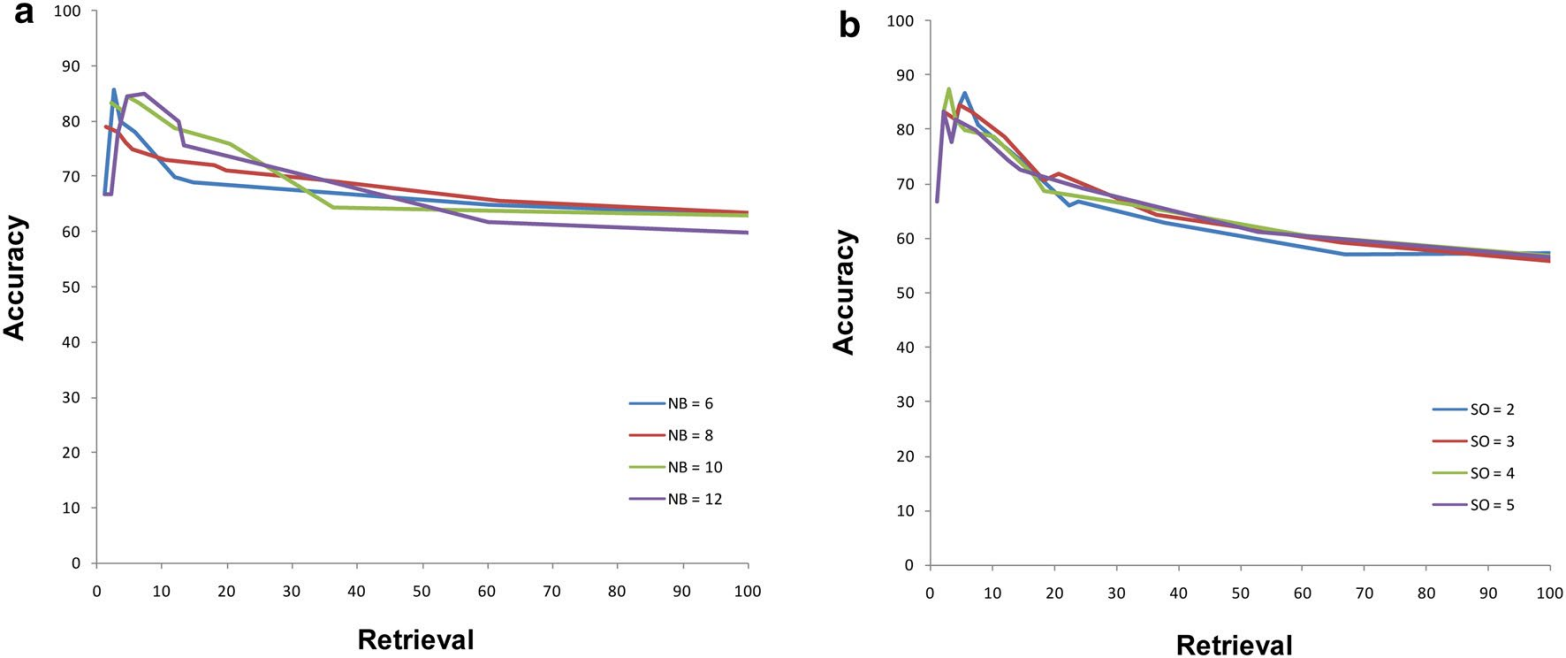
Accuracy-retrieval curves (ARCs) for different number of bins and spline orders. The relationship between accuracy and retrieval for different **a** number of bins and **b** spline orders on prostate cancer network. *NB* number of bins, *SO* spline order.

For the discretization of continuous expression data in SIREN, we tested different number of bins and the spline orders. Consistent with previous studies [27], we found the number of bins does not affect the SIREN performance remarkably as long as it is within a reasonable range (Figure 1a) and using spline order greater than three does not improve the quality of prediction significantly (Figure 1b), but rather it increases the computational cost of the algorithm.

### MI versus correlation based methods

Both MI and correlation are measures of dependency between two random variables. MI calculates the amount of information that two genes provide about each other and is always a non-negative value. Consequently, a higher mutual information value for two genes indicates that one gene is non-randomly related with the other [19]. The Pearson correlation coefficient (PCC) is a measure that indicates the intensity and trend of the linear relationship between two variables [31]. Despite the fact that the PCC can characterize linear correlations with Gaussian noise, the mutual information measure is more powerful mainly because it is able to detect non-linear dependencies that are invisible to PCC [27, 32]. Both PCC [33–37] and MI [6, 18, 27, 38–41] have been used to capture dependencies between random variables (e.g., genes).

### Benchmarks GRNs and corresponding data

To assess our algorithm, we applied SIREN to three benchmark GRNs with associated gene expression data (Additional file 2).

#### In silico constructed GRN

**Figure 2:**
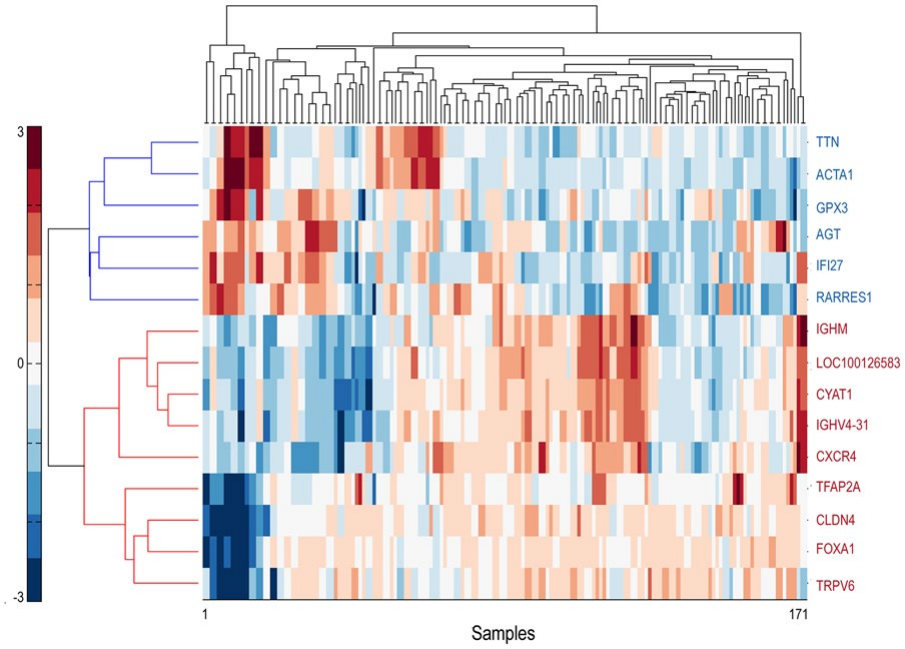
Heatmap of selected genes for the in silico network. The heatmap composed of two groups of genes. Genes in each group have similar expression pattern with each other while have opposite pattern with members of the other group. Genes in the *red* and *blue* parts are consistently up-regulated and down-regulated, respectively, during cancer progression. These groups of genes were selected based on the prostate cancer expression data.

To assess the performance of SIREN on a completely known network, we constructed an in silico GRN based on a prostate cancer gene expression data. This GRN is a clique network composed of two groups of genes. Genes in each group have a similar expression pattern to each other and opposite pattern with members of the other group (Figure 2). Because there is a clear expression pattern for each gene, we know the putative interaction types in this network by visual inspection.

#### Prostate cancer GRN

We extracted the prostate cancer GRN from the STRING functional interaction database [42], considering only regulatory interactions among genes with changed expression levels during cancer progression. To determine these genes, we focused on those which are up- or down-regulated significantly (fold change ≥2 and *p* value ≤0.05) in at least one state, considering the normal state as control. Thus the GRN is composed of genes which putatively play a role in prostate cancer and contains 1,436 interactions among 526 genes (53.8% of total genes with available transcriptome data).

#### Prostate cancer microarray data

We extracted gene expression data from the GEO database with accession number GDS2545. This dataset consists of 171 samples monitoring gene expression in four different cell states: normal prostate tissue free of any pathology (normal), normal prostate tissue adjacent to tumor (adjacent), primary prostate tumor tissue (tumor), and metastatic prostate cancer tissue (metastatic) [43].

#### *E. coli* high confidence GRN

We extracted the high confidence GRN of *E. coli* from the RegulonDB database [44]. This GRN contains 4,005 experimentally confirmed regulatory interactions among 1,696 genes. We created a sub-network from RegulonDB GRN by considering genes with available data in our transcriptomics data set. The resulting sub-network contains 2,687 interactions among 1,419 genes and was used for further analysis.

#### *E. coli* gene expression data

We extracted a microarray dataset consisting of 907 samples from the Many Microbe Microarray Database (M^3D^) Web site [45].

### SIREN algorithm

The fundamental assumption in our approach is that if two connected genes in the network have similar expression patterns, there is likely an activating (positive) association, between them. On the other hand, if their expression patterns are conversely related, the interacting genes likely have an inhibitory (negative) influence on each other.

Our method is useful for the analysis of reconstructed GRNs from any reconstruction method used to generate the input network. In addition to the GRN, SIREN also needs corresponding expression data. SIREN determines the regulation type for each pair of connected genes in the network by computing a similarity score between their expression profiles. As shown schematically in Additional file 1: Figure S2, SIREN determines the similarity between the expression profiles of two genes in four steps: (1) a B-spline discretization method is used to discretize expression data into ten bins, allowing overlap between bins to smooth the data as described in [27], (2) co-occurrence scores are calculated for each combination of bins of the genes (for example the first bin of first gene with the first bin of second gene, the first bin of first gene with the second bin of second gene, etc.) using the information-based metrics, (3) the calculated co-occurrence scores are rescaled according to the given rescaling matrix, which enables SIREN to distinguish between co-occurrences resulting from an activating or an inhibitory effect between two genes; and (4) determines the SIREN score by calculating the expected value of the rescaled co-occurrence probability scores. Mathematically speaking, the expected value of function $f(X,Y)$ is defined as: $E(f(X,Y)) = \sum\nolimits_{x,y} {p(x,y)f(x,y)}$. Therefore, SIREN score for two genes of X and Y is: $\sum\nolimits_{x,y} {p(x,y)(W(x,y)CoS(x,y))}$, where *CoS*(*x*,*y*) is the co-occurrence score, W(x,y) refers to the rescaling matrix and p(x,y) is the co-occurrence probability when X = x and Y = y.

To optimize results, we compared four distinct scoring functions and four rescaling matrices as well as tested a range of different cut-off scores for SIREN to determine a reliable threshold.

### SIREN scoring functions

To optimize the algorithm, we investigated four possible interaction scoring functions:

#### S_1_

Based on the definitions of MI and PMI, the first scoring function was defined as $S_{1} = \sum {W(x,y)} p(x,y)\log (p(x,y)/p(x)p(y))$, where *W*(*x*,*y*) is the rescaling matrix. If $g(x,y) = W(x,y)\log (p(x,y)/p(x)p(y))$, then $S_{1} = \sum {p(x,y)g(x,y)}$ or $S_{1} = E(g(x,y))$ according to the Law of the Unconscious Statistician (LOTUS) [46]. The defined *S*_1_ score is known as the weighted mutual information concept, as described in [47].

#### S_2_

To give PMI a fixed upper bound, we normalize it to have a maximum value of 1 in the case of a perfect association. The advantage of normalized PMI over PMI is that the value of PMI is usually high for rare events. It is hoped that the normalized version will reduce the low frequency bias [25]. As stated above, $PMI = \log (p(x,y)/p(x)p(y))$. In case of perfect association, we have $p(x) = p(y) = p(x,y)$, consequently $PMI = \log (p(x,y)/p(x)p(y)) = \log (p(x)/p(x)p(x)) = - \log (p(x))$.

Therefore, we can define Normalized PMI (NPMI) as

$$NPMI = \log (p(x,y)/p(x)p(y))/ - \log (p(x,y)).$$

We define the second scoring function $S_{2} = E(W(x,y) \times NPMI)$.

#### S_3_

The third scoring function is defined as $S_{3} = \sum {p(x,y)[W(x,y)]}$. This score measures the expected value of the rescaling matrix *W*(*x*,*y*).

#### S_4_

In a similar approach to the second scoring function, to give MI a fixed upper bound, we normalize MI to have a maximum value of 1 in the case of a perfect association [25]. As above, $MI = \sum {p(x,y)\log (p(x,y)/p(x)p(y))}$. If $p(x) = p(y) = p(x,y)$, then $MI(x,y) = MI(x,x) = \sum {p(x,x)\log (p(x,x)/p(x)p(x))}$. Consequently, $MI(x,x) = \sum {p(x,x)( - \log (p(x)))} = - \sum {p(x)\log (p(x))}$. Therefore $Normalized\;MI(NMI) = {{\sum {p(x,y)} (\log (p(x,y)/p(x)p(y))} \mathord{\left/ {\vphantom {{\sum {p(x,y)} (\log (p(x,y)/p(x)p(y))} { - \sum {p(x,y)\log (p(x,y))} }}} \right. \kern-0pt} { - \sum {p(x,y)\log (p(x,y))} }}$.

We defined *Normalized Rescaled MI* (*NRMI*) as $S_{4} = {{\sum {p(x,y)\left( {W(x,y)\left( {\log (p(x,y)/p(x)p(y))} \right)} \right)} } \mathord{\left/ {\vphantom {{\sum {p(x,y)\left( {W(x,y)\left( {\log (p(x,y)/p(x)p(y))} \right)} \right)} } { - \sum {p(x,y)} \left( {W(x,y)\left( {\log (p(x,y)} \right)} \right)}}} \right. \kern-0pt} { - \sum {p(x,y)} \left( {W(x,y)\left( {\log (p(x,y)} \right)} \right)}}$.

### SIREN performance assessment

To measure SIREN performance, we defined two measures: true number (TNu) and false number (FNu). positive true positive (PTP) and negative true negative (NTN) is the number of interactions correctly signed positive and negative, respectively, whereas negative false positive (NFP) and positive false negative (PFN) is the number of interaction types that SIREN has assigned incorrectly positive and negative, respectively. TNu is the number of PTP plus the number of NTN, while FNu is the number of NFP plus the number of PFN. Accuracy, defined as TNu/(TNu + FNu), is the fraction of correctly signed interactions among all interactions signed by SIREN, while retrieval is the number of regulatory interactions signed by SIREN among all interactions (some of which are not signed). Performance of the algorithm is assessed using accuracy-retrieval curves.

## Results and discussion

### Determining the interaction types in GRNs

MI is used extensively for reconstructing GRNs because it has a low computational complexity and is able to capture nonlinear dependencies among variables [48–50]. However, using MI has some disadvantages, including that it does not reveal the interaction type between two random variables. In the context of GRN reconstruction, this means that it does not identify whether a regulatory interaction is positive (activating) or negative (inhibiting) to overcome this challenge, we modified the conventional mutual information formula by adding a rescaling matrix. This rescaling matrix converts the MI function, which normally generates a non-negative score to a function that can have negative values. The resulting sign is used to predict the interaction type.

Using expression data, our new method can determine the interaction type between two interacting genes in a GRN. SIREN discriminates activating from inhibitory associations based on the premise that the effect of two interacting genes on each other will be reflected in their expression patterns across multiple cellular conditions. Similar expression profiles indicate a positive interaction and dissimilar profiles indicates a negative interaction.

### Selecting the optimum interaction scoring function

**Figure 3:**
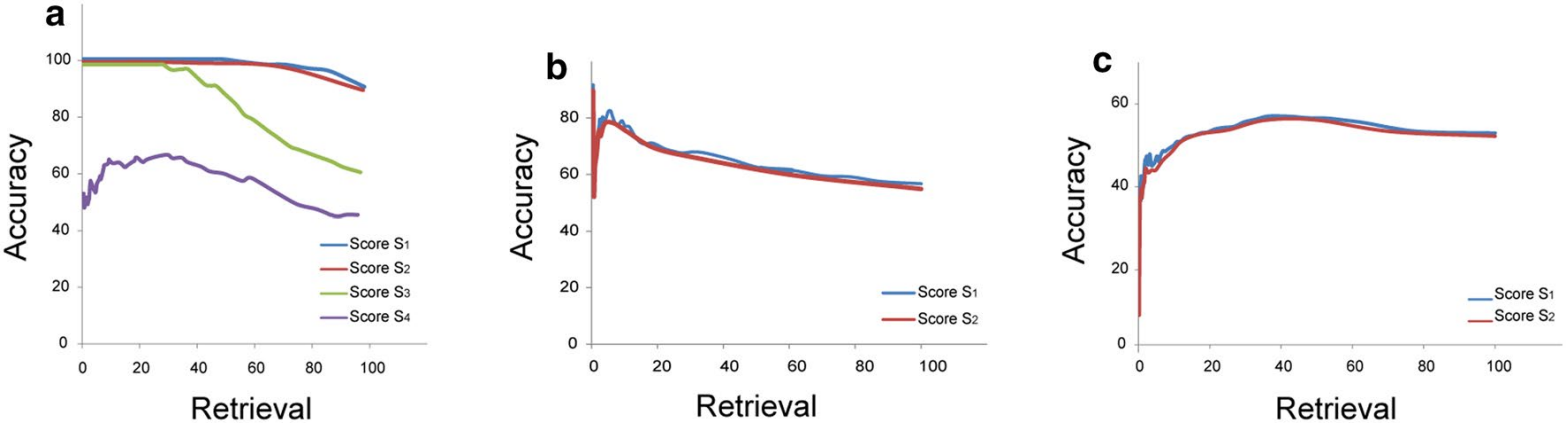
Comparison of four possible scoring functions. *Plots* compare four different scoring functions on three different GRNs: **a** in silico constructed GRN with four possible scoring functions, **b** prostate cancer GRN with S_1_ and S_2_, and **c** *E. coli* high confidence GRN with S_1_ and S_2._

To optimize SIREN, we defined four different scoring functions as well as four different rescaling matrices. To evaluate the performance of our algorithm in each case, we considered three different GRNs: an in silico constructed GRN, a prostate cancer GRN, and an *E. coli* high confidence GRN. The results, averaged across the four possible rescaling matrices, are presented in Figure 3.

#### In silico GRN

**Figure 4:**
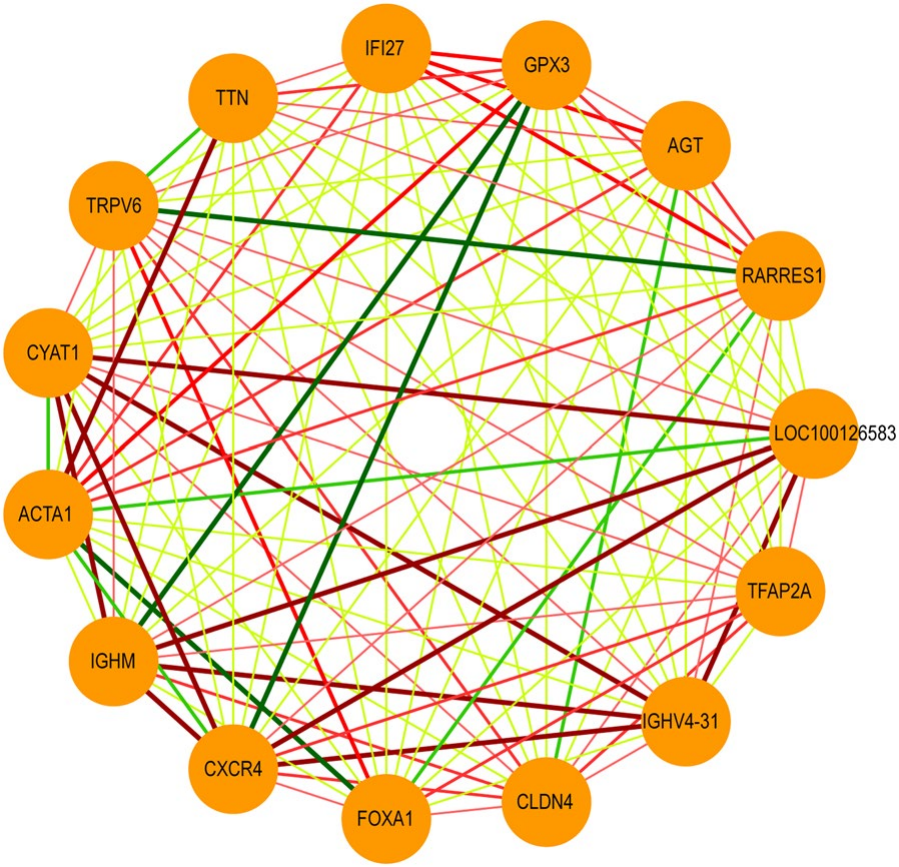
The in silico network. The constructed network clique is visualized with Cytoscape [51] (version 3.0.0). *Red* and *green edges* indicate positive and negative interaction types, respectively. *Edge width* shows the SIREN score for each edge.

To evaluate the performance of SIREN algorithm, we selected nine (*CXCR4*, *IGHM*, *TFAP2A*, *IGHV4*-*31*, *CLDN4*, *LOC100126583*, *CYAT1*, *TRPV6*, *FOXA1*) and six (*IFI27*, *RARRES1*, *GPX3*, *AGT*, *ACTA1*, *TTN*) genes that were consistently up-or down- regulated during cancer progression, respectively (Figure 2). We then generated a clique network consisting of all possible interactions between these genes (105 interactions). This network consists of 51 positive (up–up or down–down) and 54 negative (up–down) interactions (Figure 4). Application of SIREN on the in silico network, as shown in Figure 3a, indicated that the scoring functions S_1_ and S_2_ have comparable performance while out-performing S_3_ and S_4_. Closer examination of the results showed that S_3_ is not sensitive enough to detect negative interactions because negative interactions are often derived from small magnitudes of the changes in expression levels. The logarithmic component of the S_1_ and S_2_ scoring functions magnifies these small differences in expression levels, but this is not present in S_3_. S_4_ had the lowest accuracy. Hence, we selected S_1_ and S_2_ scoring functions for further evaluations.

#### Prostate cancer GRN

STRING is a functional interaction database that includes regulatory interactions. We extracted a functional interaction network based on genes that show alteration in their expression profile during prostate cancer progression, limited to regulatory interactions which we define as the prostate cancer GRN (see “Methods” for details). The extracted GRN was composed of 1,436 interactions among 526 genes (102 negative, 176 positive and 1,158 interactions with no sign). We then ran SIREN on this network and compared SIREN’s predicted interaction type to the known interaction type from STRING. As shown in Figure 3b, both S_1_ and S_2_ scoring functions had, again, comparable results on this GRN with about 70% accuracy.

#### *E. coli* GRN

We applied SIREN to an experimentally constructed GRN of *E. coli* containing 1,408 positive and 1,279 negative interactions. As shown in Figure 3c, both scoring functions S_1_ and S_2_ resulted in similar accuracies (56% maximum). In this test, the experimentally constructed GRN of *E. coli* and the gene expression data where from independent sources. The low performance of SIREN in this case is most probably because some regulatory circuits may not be reflected in the gene expression data. As an illustration, for two genes that have negative regulation on each other via negative feedback loop, we expect to observe that up-regulation of the regulator leads to the down-regulation of the regulated gene. However, if the expression level of regulator gene does not increase perceptibly, the expression patterns will not reflect the inhibitory effect. Thus, both genes will show similar expression patterns, and consequently, an activating interaction will be wrongly inferred. Considering this fact, our approach can reliably detect interaction types only for genes that show some level of alteration in the expression in the corresponding expression data set. Consistently, we found restricting the *E. coli* GRN to 10% most fluctuated genes (this sub-network was composed of 31 positive and 13 negative interactions) resulted in the precision of 68.18% (22 PTP and 8 NTN) for SIREN algorithm.

**Figure 5:**
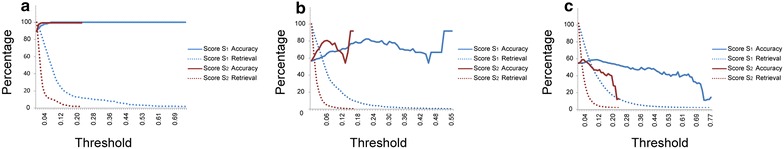
Comparing S_1_ and S_2_ scoring functions. *Plots* compare accuracy and retrieval of S_1_ and S_2_ scoring functions on three different GRNs: **a** in silico constructed GRN, **b** prostate cancer GRN, and **c** *E. coli* high confidence GRN. The *x-axis* represents threshold (0 to ±1) and the *y-axis* shows accuracy and retrieval percentage.

Scores obtained from the S_1_ scoring function have a wider range compared with the scores of S_2_ scoring functions in all three GRNs (Figure 5). Also, the S_1_ scoring function has a lower computational complexity. Considering these points, we selected S_1_ as the best scoring function for further investigations.

### Selecting the optimum rescaling matrix

**Figure 6:**
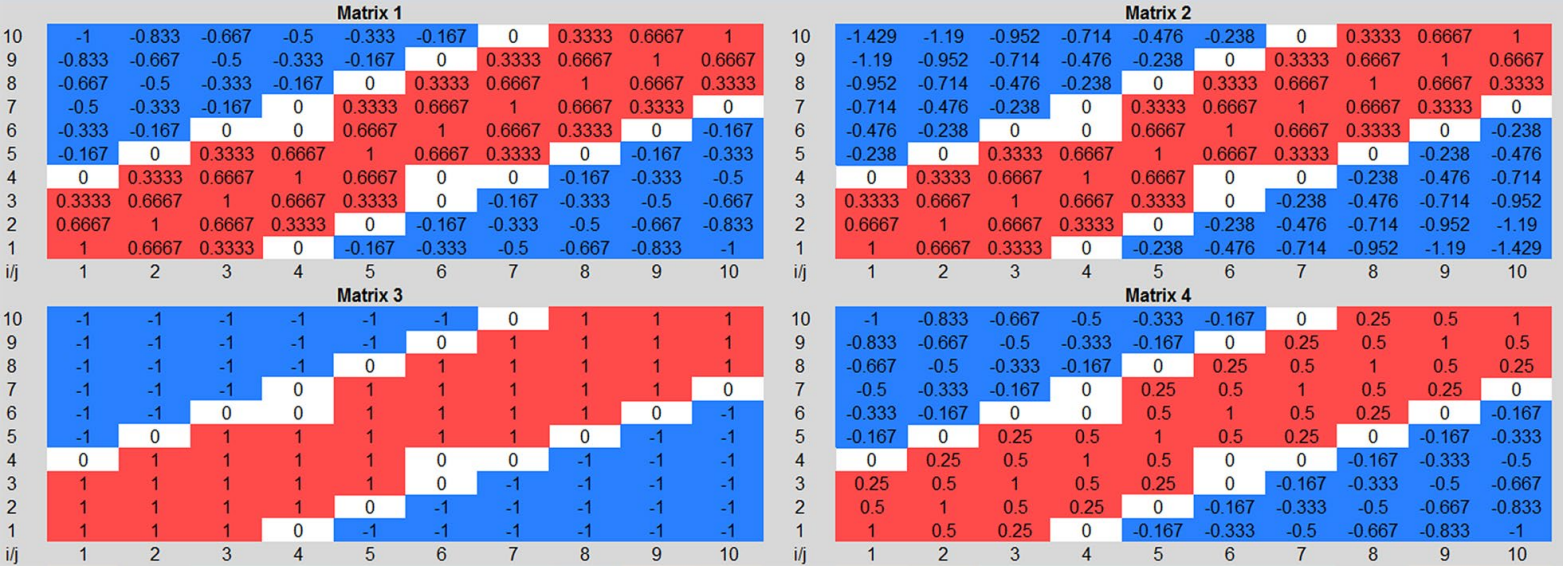
Four rescaling matrices. The design of four rescaling matrices evaluated for use in SIREN.

We have used a rescaling matrix to convert the MI function, which normally generates a non-negative score, to a function that can produce negative values. The resulting sign is used to predict either an activating (similar expression profiles) or inhibitory effect (different expression profiles) between genes. Using the B-spline approach, we smoothly discretized the expression profile of each gene into 10 bins. For each interacting pair, SIREN creates a two-dimensional grid with 100 cells. The distribution pattern of expression data in these 100 cells is used for predicting the interaction type. The interaction type can be inferred from this grid because the distribution pattern for genes with positively correlated expression patterns will be different from the distribution pattern of genes with a negative association. To discriminate the distribution patterns from each other, we have introduced the rescaling matrix (Figure 6). The design of four rescaling matrices evaluated for use in SIREN. For all the matrices, we initially assigned −1 to the two most negative bins and +1 to the most positive bins, on the diagonal of the matrix. We also arranged the matrices to each have equal number of positive and negative cells (42 positive, 42 negative, and 16 zero cells).

For matrix 1, for the other negative bins, we repeatedly subtracted 0.166667 (one divided by six negative levels) as we moved towards the border between positive and negative. The positive cells were treated similarly. The positive cells in matrix 2 are the same as in matrix 1; however, to improve detection of negative interactions, and to compensate for the +10 discrepancy in overall weight arising from the matrix’s diagonal, we increased the weight on the negative cells such that the overall weight in the matrix was equal to zero. For matrix 3, we simply assigned −1 and +1 to each non-zero cell. The negative cells in matrix 4 are the same with matrix 1; for the positive cells, we used a multiplicative scaling factor rather than an additive one.

**Figure 7:**
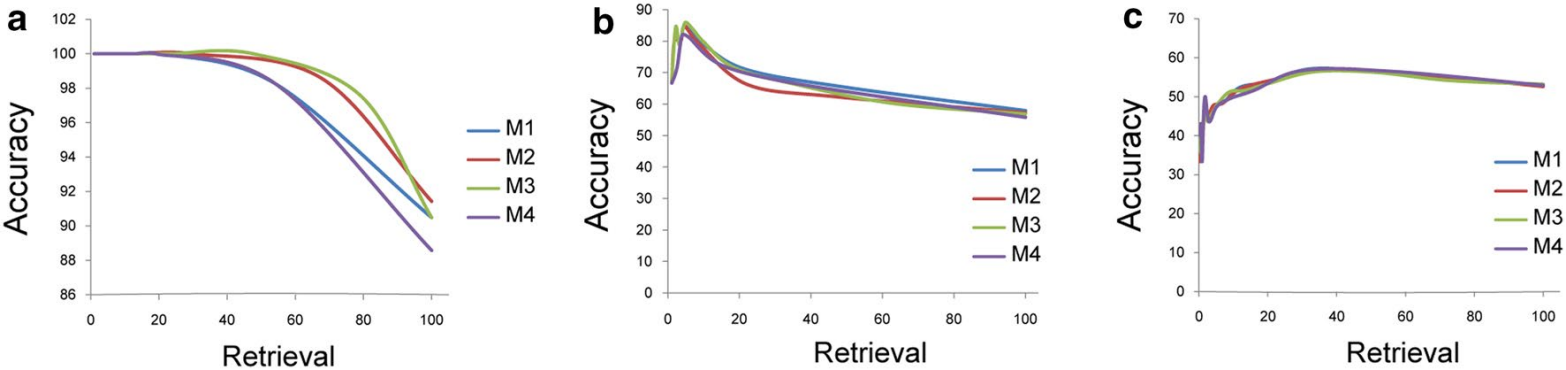
Evaluation of four rescaling matrices. Three plots show the result of applying SIREN using scoring function S_1_ with each rescaling matrix on three GRNs. **a** The M_3_ rescaling matrix performs best with the in silico network. **b**, **c** SIREN is robust to choice of rescaling matrix for experimentally derived GRNs.

To select the best performing matrix from these four matrices, we examined the performance of each of them on the three selected benchmark GRNs (Figure 7). Our results indicate that SIREN is robust to the selected rescaling matrix, especially for the experimentally derived GRNs. However, the M_3_ rescaling matrix (Matrix 3) (Figure 6) performs better with the in silico network (Figure 7). This may be caused by the higher ratio of negative interactions in the in silico network (51.43%). Considering results obtained from this step, we chose M_3_ as the optimum rescaling matrix.

### Selecting the best threshold

**Figure 8:**
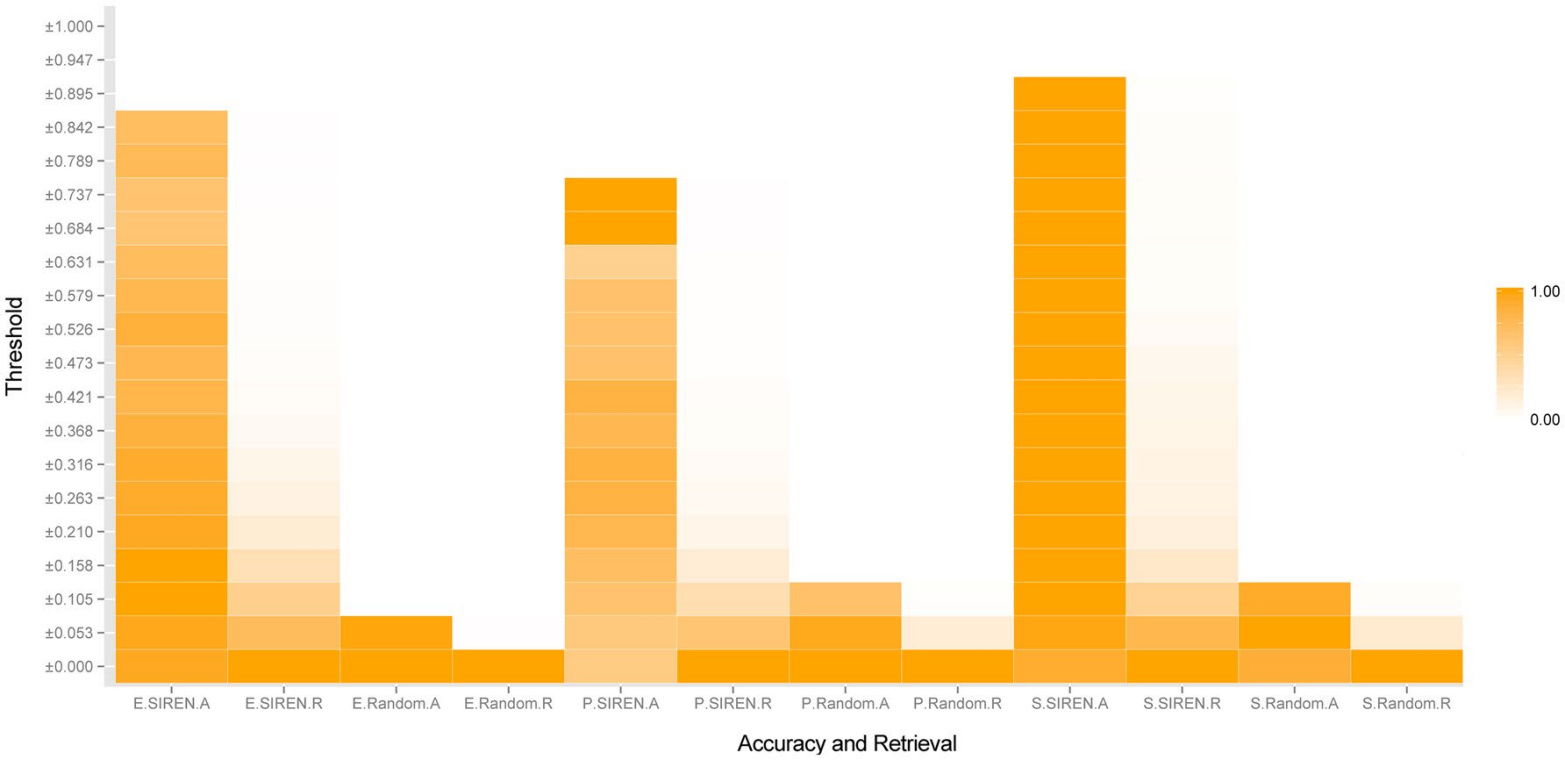
Selecting the best threshold for SIREN. The relationship between accuracy and retrieval and the best threshold with highest accuracy and fair retrieval. *A* accuracy, *R* retrieval; *E* *E. coli*, *P* prostate, *S* in silico. As the *figure* shows in ±0.158, no interaction type was detected randomly by SIREN where the color spectrums were changed to be completely white in accuracy and retrieval of all three random networks.

To select the best threshold on the resulting SIREN score, we applied it to the *E. coli*, prostate and in silico benchmarks, using the S_1_ scoring function and the M_3_ rescaling matrix. We tested a range of different cut-off scores (20 different thresholds between 0 and 1) for SIREN to determine a reliable threshold for various networks. The results showed that when the cut-off threshold is greater than +0.158 or smaller than −0.158, SIREN does not detect any interaction type in random data (generated by 10^6^ times of shuffling gene expression data) (Figure 8), while many interactions are predicted to be signed in the benchmark GRNs.

**Figure 9:**
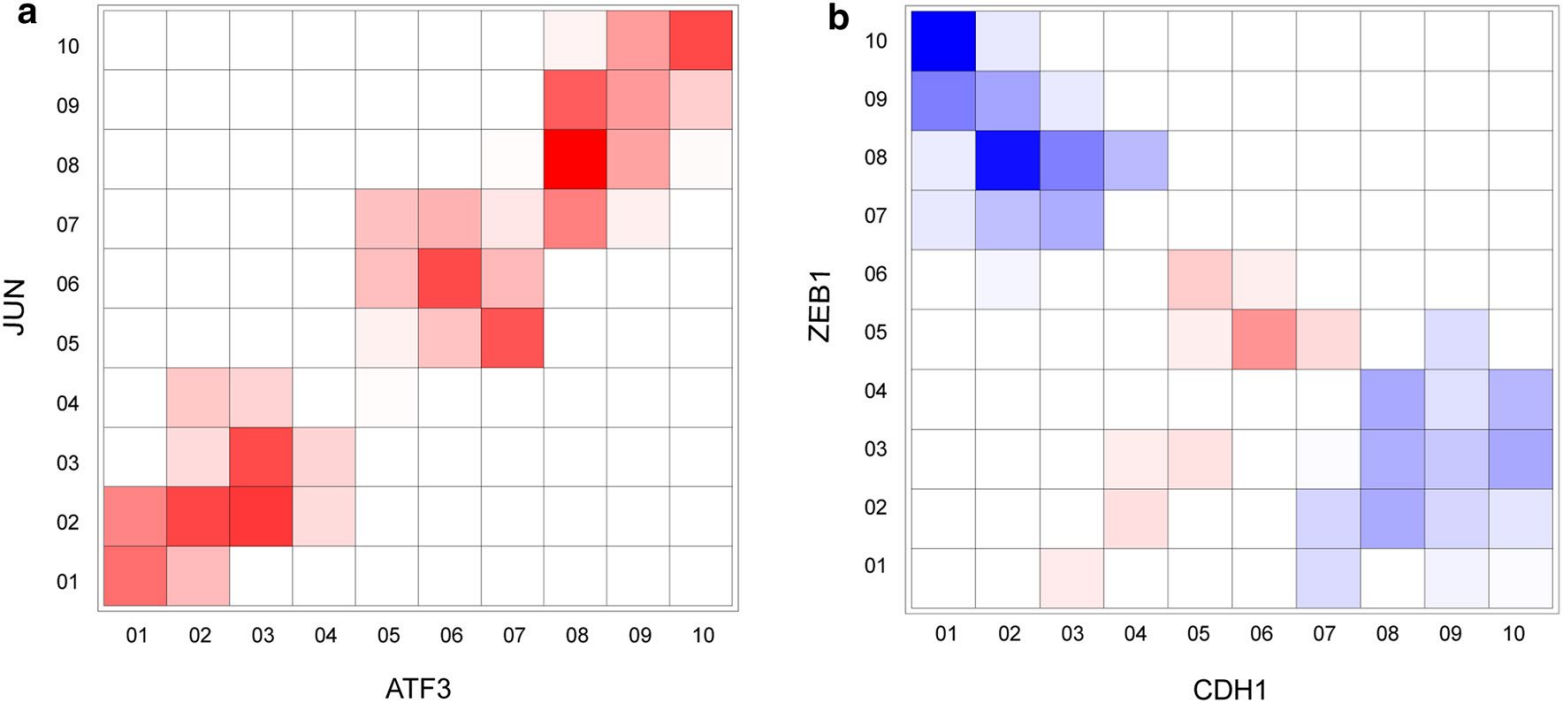
Deciphering interaction type from co-expression patterns. **a**, **b** The two-dimensional grids are constructed for two selected gene pairs with known activating or inhibitory effect on each other. The *color density* for each cell in the grid represents the computed PMI for that cell multiplied in the occurrence probability of the cell and corresponding rescaling value (determined based on the rescaling matrix). The PMI and occurrence probability is calculated based on the associated transcriptome data. *Red* indicates positive score and *blue* represents negative score (defined based on the M_3_ rescaling matrix). SIREN score is determined by summing up the calculated values for each combination of bins. **a** For two example genes with known activating relationships (*JUN* and *ATF3*), cells defined as activating have non-zero values and cells defined as inhibitory relationship, have zero values. **b** This situation is reversed for two genes with known inhibitory relationship (*ZEB1* and *CDH1*).

Figure 9 demonstrates the application of SIREN (with optimized parameters) on two example interactions, one activating and one inhibitory. As mentioned earlier, SIREN detects the interaction type in four steps: (1) discretizing the expression profile of each gene into 10 bins; (2) calculating the co-occurrence probability for each combination of bins using the PMI metric; (3) defining an activating or inhibitory relationship for each combination of bins by aid of a rescaling matrix; and (4) calculating a final score by integrating the calculated values for each combination of bins. For example, with two genes with a known activating relationship from STRING (*JUN* and *ATF3*) [52, 53], bins defined as activating have non-zero values while bins defined as inhibitory have mostly zero values (Figure 9a). This situation is reversed for two genes with a known inhibitory relationship (*ZEB1* and *CDH1*) [54] (Figure 9b).

### Deficiency in current knowledge about interaction types

**Figure 10:**
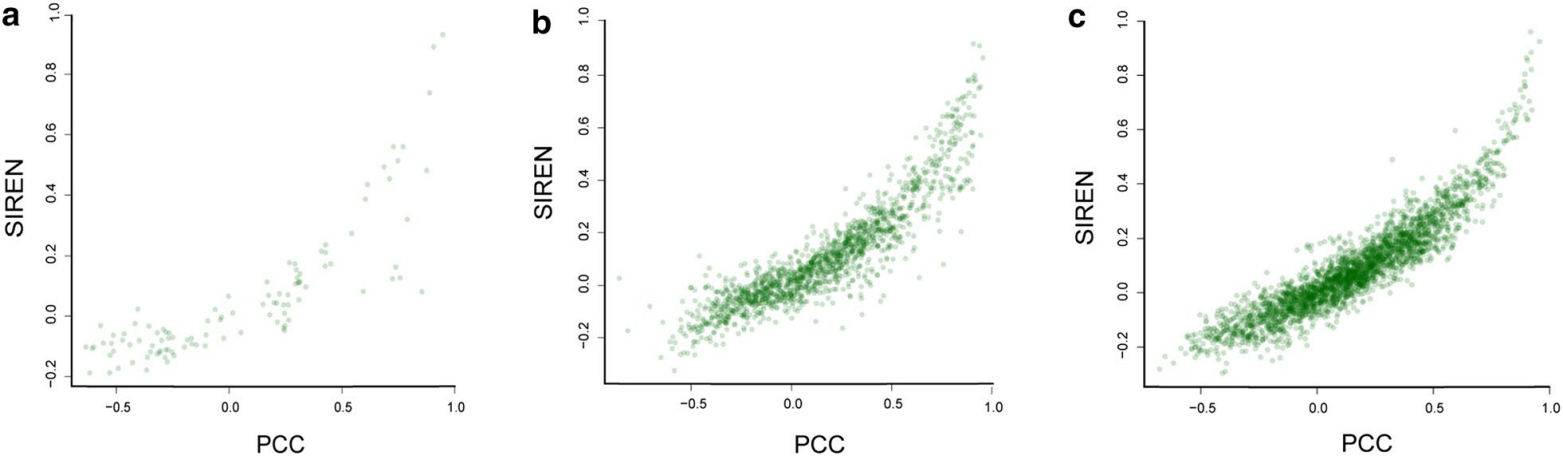
Close relationship between Pearson correlation coefficient and SIREN. *Three figures* illustrate the relationship between PCC and SIREN results for three GRNs: **a** in silico GRN, **b** prostate cancer GRN, and **c** *E. coli* high confidence GRN.

**Figure 11:**
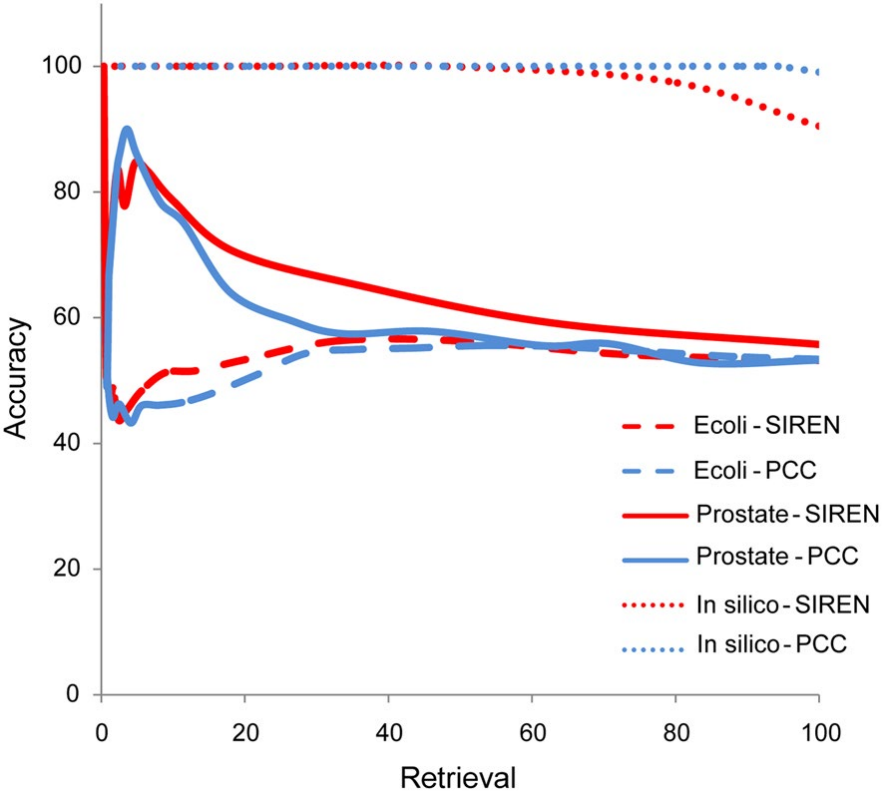
SIREN versus Pearson correlation coefficient. The relationship between accuracy and retrieval of SIREN was compared with PCC on three GRNs.

PCC has been widely used to decipher the interaction type based on transcriptome data [55, 56]. We compared SIREN with PCC by applying both to all three GRNs. This comparison revealed that their overall results are similar, suggesting that most regulatory associations in the considered GRNs have a linear or monotonic nature (Figure 10). However, the results of PCC and SIREN are inconsistent for some interactions. For example two genes with an activating relationship from STRING (*EGR1* and *FGF2*) [57] are determined to have an inhibition relationship using PCC while SIREN inferred a positive association. On the other hand *MICA* and *IL10* show an inhibition association [58] by STRING and SIREN; while they have positive association based on PCC. Ultimately, SIREN shows superior performance in all but the in silico network case (Figure 11), indicating that consideration of non-linear relationships in the gene expression data is useful.

Our SIREN predictions on the prostate cancer GRN included 454 interactions for which no regulatory type existed in STRING (Additional file 3). A literature search on a sample of these newly signed GRN supports the reliability of our results. For example, we predict a positive association between *EGR1* and *ATF3* genes, which is in line with previous studies that have shown these two genes associated with each other and *ATF3* is a target of *EGR1* that induces an up-regulation of *ATF3* [59].We also found a negative association between *FASN* and *CAV*-*1* which is consistent with previous reports that showed *FASN* interact with *CAV*-*1*, a marker for metastasis state of prostate cancer, and inactivation of *CAV*-*1* mediates by *FASN* [52].

## Conclusion

At present, there is no information theory-based framework to detect regulatory interaction types in gene regulatory networks. In this work, we tried to fill this gap by exploiting the notion that the effect two interacting genes have on each other can be observed in their expression patterns. This idea allowed us to develop an information theory-based solution, SIREN, to identify interaction types using gene expression data. SIREN increases the amount of information available for GRNs compared to standard GRN inference algorithms. SIREN runs reasonably fast; computing the 2,687 interactions among 1,419 genes took 6 min on an Intel Core i5 system with 4 GB of RAM; hence it is usable with large biological networks. Additionally, we have shown the method is applicable to prokaryotic and eukaryotic GRNs.

## Additional files

Additional file 1: The B-spline function and SIREN algorithm schematically. This file contains the additional figure S1 and S2. In figure S1, data are discretized to 4 bins (Lowest, Low, High, and Highest) and the B-spline order is equal to 3. As shown, bins have overlap with each other. The dashed line represents a sample data point. The place that dashed line intersect with each bin, determines the weight of that bin. Note that sum of weights is always equal to one. In figure S2 given a GRN and corresponding transcriptome dataset, SIREN starts with discretization of continuous transcriptome data. Next, SIREN constructs a co-occurrence profile for each interacting genes in the GRN. Based on the patterns of co-occurrence, SIREN determines the type of interaction (Inhibitory or activatory). Additional file 2: Signed GRNs. The list of benchmark GRNs contains E. coli high confidence, Prostate cancer, and in silico constructed. Additional file 3: Prostate cancer GRN. 454 SIREN predicted interactions, not present in the STRING database.

## Abbreviations

ARCs: accuracy-retrieval curves
FNu: false number
GRNs: gene regulatory networks
LOTUS: Law of the Unconscious Statistician
M^3D^: many microbe microarray database
MI: mutual information
NB: number of bins
NFP: negative false positives
NMI: normalized MI
NRMI: normalized rescaled MI
NTN: negative true negatives
PCC: Pearson coefficient correlation
PFN: positive false negatives
PMI: point-wise mutual information
PTP: positive true positives
SIREN: signing of regulatory networks
SO: spline order
TNu: true number
