# Erratum to: Inferring interaction type in gene regulatory networks using co-expression data

**DOI:** 10.1186/s13015-015-0055-3

**Published:** 2015-08-11

**Authors:** Pegah Khosravi, Vahid H Gazestani, Leila Pirhaji, Brian Law, Mehdi Sadeghi, Gary D Bader, Bahram Goliaei

**Affiliations:** School of Biological Sciences, Institute for Research in Fundamental Sciences (IPM), Tehran, Iran; The Donnelly Centre, University of Toronto, Toronto, Canada; Institute of Parasitology, McGill University, Montreal, QC Canada; Department of Biotechnology, College of Science, University of Tehran, Tehran, Iran; Department of Computer Science, University of Toronto, Toronto, Canada; National Institute of Genetic Engineering and Biotechnology, Tehran, Iran; Department of Bioinformatics, Institute of Biochemistry and Biophysics (IBB), University of Tehran, Tehran, Iran

## Erratum to: Algorithms Mol Biol (2015) 10:23 DOI 10.1186/s13015-015-0054-4

In the original publication of this article [1], the sequence of authors in the author list was published incorrectly. The correct author list is included above and is as follows:

Pegah Khosrav, Vahid H Gazestani, Leila Pirhaji, Brian Law, Mehdi Sadeghi, Gary D Bader and Bahram Goliaei.

In addition, Pegah Khosravi and Gary D Bader were both listed as joint corresponding authors of the article, however this was incorrect. Pegah Khosravi is the sole corresponding author for the article.

Please note the affiliations for Brian Law and Gary D Bader were amended as above.
