# Supplementary material for: Inferring interaction type in gene regulatory networks using co-expression data
Source: Algorithms Mol Biol. 2015 Jul 8;10:23. doi: 10.1186/s13015-015-0054-4 (PMC4495944; doi:10.1186/s13015-015-0054-4)
Supplement: Additional file 1: — The B-spline function and SIREN algorithm schematically. This file contains the additional figure S1 and S2. In figure S1, data are discretized to 4 bins (Lowest, Low, High, and Highest) and the B-spline order is equal to 3. As shown, bins have overlap with each other. The dashed line represents a sample data point. The place that dashed line intersect with each bin, determines the weight of that bin. Note that sum of weights is always equal to one. In figure S2 given a GRN and corresponding transcriptome dataset, SIREN starts with discretization of continuous transcriptome data. Next, SIREN constructs a co-occurrence profile for each interacting genes in the GRN. Based on the patterns of co-occurrence, SIREN determines the type of interaction (Inhibitory or activatory). [file 13015_2015_54_MOESM1_ESM.pdf]

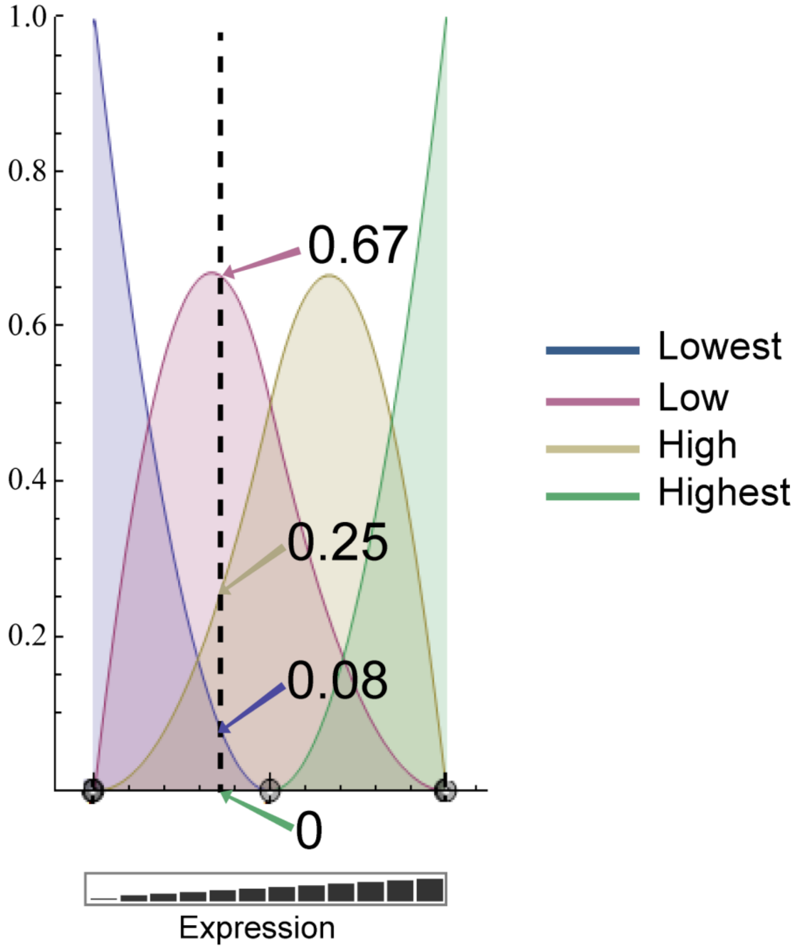

Figure S1. Schematic representation of B-spline discretization method. In this figure, data are discretized to 4 bins (Lowest, Low, High, Highest) and the B-spline order is equal to 3. As shown, bins have overlap with each other. The dashed line represents a sample data point. The place that dashed line intersect with each bin, determines the weight of that bin. Note that sum of weights is always equal to one.

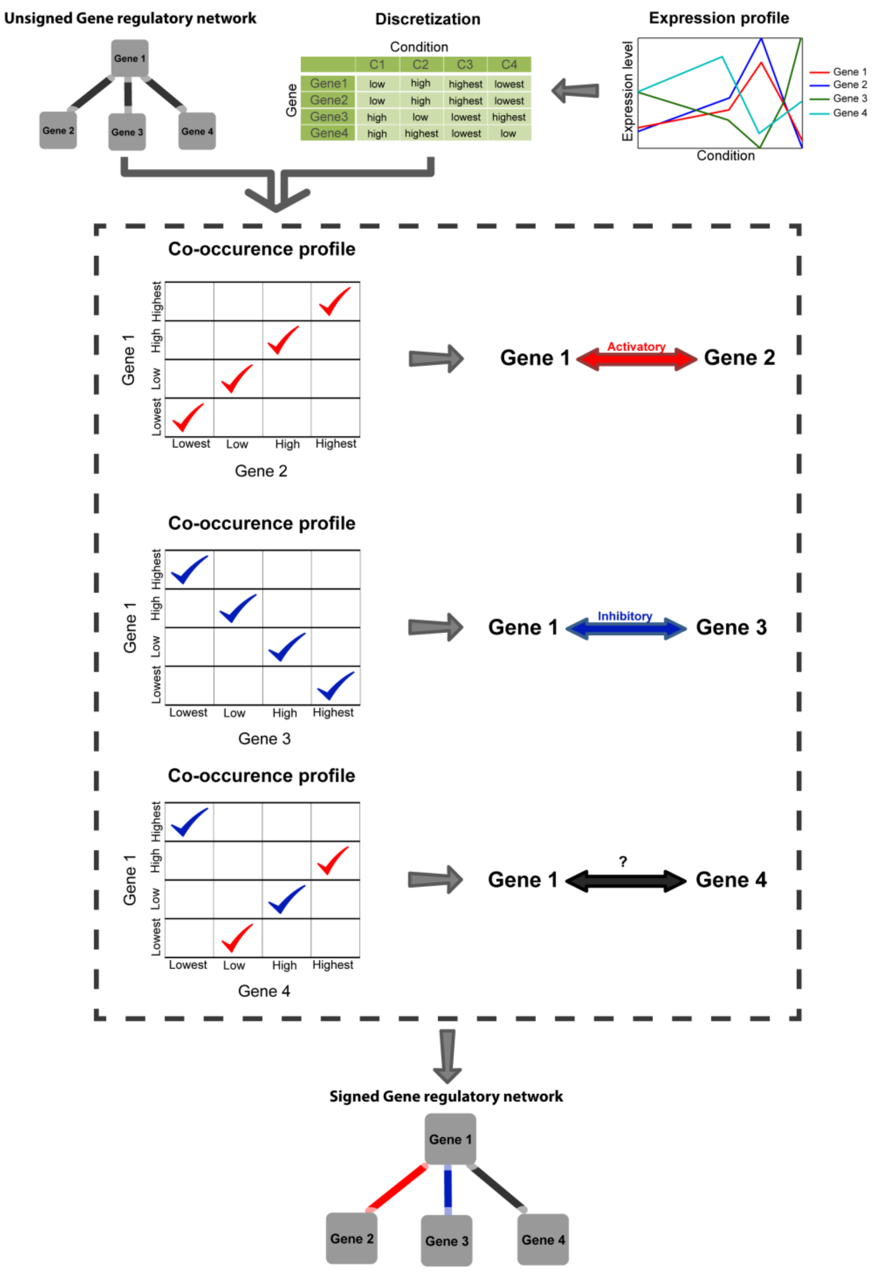

Figure S2. Schematic representation of SIREN algorithm. Given a GRN and corresponding transcriptome dataset, SIREN starts with discretization of continuous transcriptome data. Next, SIREN constructs a co-occurrence profile for each interacting genes in the GRN. Based on the patterns of co-occurrence, SIREN determines the type of interaction (Inhibitory or activatory).
